# Predictors of female sexual problems in Shanxi, China: a population-based cross-sectional epidemiologic survey

**DOI:** 10.1093/sexmed/qfac005

**Published:** 2023-01-12

**Authors:** Duo Yuan, Xian-hui Zhang, Jie Pan, Ying-an Zhang, Zhao-ai Li, Xiao-li Li

**Affiliations:** Department of Obstetrics and Gynecology, Shanxi Children’s Hospital, Shanxi Maternal and Child Health Hospital, Taiyuan, China; Department of Obstetrics and Gynecology, The Second Hospital of Shanxi Medical University, Taiyuan, China; Department of Internal Medicine, Shanxi Children’s Hospital, Shanxi Maternal and Child Health Hospital, Taiyuan, China; Department of Pathology, Stanford University School of Medicine, CA, 94305, United States; Department of Obstetrics and Gynecology, Shanxi Children’s Hospital, Shanxi Maternal and Child Health Hospital, Taiyuan, China; Department of Obstetrics and Gynecology, Shanxi Children’s Hospital, Shanxi Maternal and Child Health Hospital, Taiyuan, China; Department of Obstetrics and Gynecology, Shanxi Children’s Hospital, Shanxi Maternal and Child Health Hospital, Taiyuan, China

**Keywords:** female sexual problems, female sexual function index, Chinese version of the female sexual function index, risk factor, Shanxi

## Abstract

**Background:**

Large studies on female sexual function have been conducted globally. Nonetheless, whether the state of female sexual function in China is significantly different from that in the rest of the world is largely unknown.

**Aim:**

In this study, we aimed to investigate the associated risk factors for sexual problems in women in Shanxi, China, by conducting a population-based cross-sectional epidemiological survey.

**Methods:**

Using the Chinese version of the Female Sexual Function Index (CV-FSFI), we surveyed women aged 20-70 years to diagnose the sexual problems. We used multiple linear regression models to estimate the risk factors for sexual problems.

**Outcomes:**

We used the CV-FSFI for investigating the female sexual function.

**Results:**

Our results included 6720 women, of whom 1205 were the sexually inactive and 5515 were sexually active. The mean FSFI score for sexually active women was 25.38 ± 4.20 (99% CI 25.27-25.49). Negative numerical coefficients were found for model predictors of age (*B* = −0.134, *P* < 0.001), postmenopausal status (*B* = −2.250, *P* < 0.001), chronic diseases (*B* = −0.512, *P* < 0.001), and gynecologic diseases (*B* = −0.767, *P* < 0.001). In contrast, positive numerical coefficients were found for education (*B* = 0.466, *P* < 0.001) and cesarean section (*B* = 0.312, *P* = 0.009).

**Clinical Implications:**

It is important to pay attention to the sexual health of women and explore the factors influencing the sexual problems of women in China.

**Strengths and Limitations:**

The present study is to our knowledge the first to evaluate the sexual function of women in Shanxi, China. Answers to questions asked in the CV-FSFI survey may be somewhat subjective, and thus additional tools and documentation are probably needed for accurate assessment.

**Conclusion:**

Similarly to other worldwide studies, our study found that increasing age, postmenopausal status, chronic diseases, and gynecological diseases were risk factors for sexual problems, whereas high education levels and cesarean section childbirth were protective factors for sexual problems.

## Introduction

Sexuality is a significant component in the life of every woman.^1^ Female sexual dysfunction (FSD) is an essential public health problem that adversely affects the well-being of women by causing a range of physical, psychological, and social disorders.^2^ According to the Diagnostic and Statistical Manual of Mental Disorders V (DSM-V) of the American Psychiatric Association, FSD includes female sexual interest/arousal disorder (FSIAD), female orgasmic disorder (FOD), and genito-pelvic pain/penetration disorder (GPPD).^3–5^ FSD is a common problem affecting women’s quality of life and family happiness.^6^ Culture, education, and religion influence beliefs about female sexuality. Most women ignore their sexual status and do not actively seek medical treatment when sexual problems occur.^7^ Berman et al.^8^ reported that 40% of women did not ask for help from a doctor for their sexual disorder.

There are several methods to assess female sexual function. The Female Sexual Function Index (FSFI) is a patient self-report scale developed by Rosen et al. in 2000 to assess the sexual function of heterosexual women during the past 4 weeks.^9–11^ This scale has been translated into Chinese, Korean, and other languages that are widely used in the international community.^12^ For a sexual problem to be considered a sexual dysfunction, it must be accompanied by distress. It is worth noting that FSFI, as a screening tool, cannot be used to diagnose FSD because the FSFI does not assess the distress component. Nonetheless, this scale is still recommended because a low score indicates a greater likelihood of FSD. The Chinese version of the FSFI (CV-FSFI) is used to assess the risk of female sexual problems in China. Evaluations done by use of the CV-FSFI in China have been reliable and effective.^13^

Sexual health is vital for overall health and well-being.^14^ Thus, it is crucial to explore the associated risk factors as part of a strategy for conducting early intervention and treatment of sexual problems in women.^15^ Availability of more data on sexual function would help increase understanding of the burdens caused by sexual problems. Additionally, identification of the risk factors for sexual problems aids in the development of preventable strategies to enhance quality of life in women. Due to social changes in recent decades, Chinese women acquire a tremendous amount of sexual knowledge.^16^ Numerous studies on female sexual function have been conducted worldwide.^17^ However, since Chinese women are often reluctant to discuss sexual topics publicly, it is difficult to collect data on female sexual function in China. Whether the state of female sexual function in China is significantly different from that in the rest of the world is largely unknown. The purpose of this study was to investigate the risk factors associated with sexual problems in women in Shanxi, China.

## Methods

### Sampling

This cross-sectional study was part of a nationwide study on pelvic floor dysfunction in Chinese women. The study was carried out in 6 areas that represented the geographical and economic regions of Shanxi. A stratified random sampling method was used to perform the quantitative phase of the study.

**Figure 1:**
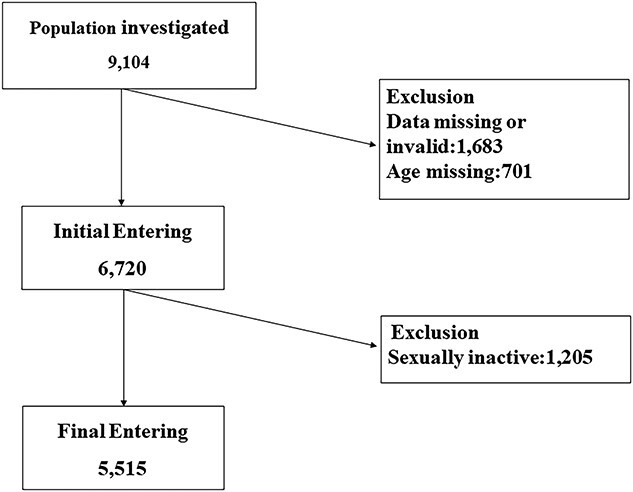
Study flow diagram.

The study was monitored by the institutional review board at the top research site and consisted of members from the Peking Union Medical College Hospital, Chinese Academy of Medical Science, Beijing, China. The survey was conducted by Shanxi Children’s Hospital, Shanxi Maternal and Child Health Hospital. The participants were women in the age group of 20–70 years. All eligible women were invited to participate in the screening. The investigators received professional training and passed an examination upon completion. Investigators were required to respect the privacy of the respondents and communicate with respondents to gain their trust. All participants provided written informed consent. The questionnaires were distributed at local community health centers by medical staff trained in data collection. The respondents filled out the questionnaire anonymously and independently. In all, 9104 women completed a questionnaire on sexual function. After excluding missing and invalid data, 6720 women were enrolled (Figure 1). Finally, 5515 women who reported an active sex life in the last 4 months were enrolled. The Ethics Committee of Shanxi Children’s Hospital, Shanxi Maternal and Child Health Hospital approved the study.

### Measures

In the current study, we used the CV-FSFI for investigating the female sexual function in Shanxi, China. The CV-FSFI consists of 19 questions covering 6 domains, including desire, arousal, lubrication, orgasm, satisfaction, and pain. For each question, the scores range from 0 to 5 to represent the variation in frequency, intensity, and satisfaction of each domain. A particular domain score is the sum of the scores of individual questions multiplied by the factor specific to the relevant domain. The total score is the sum of the scores of the 6 domains. The total score ranges from 2.0 to 36.0, with lower scores indicating a greater degree of sexual problems.^18^

Responses to each question were reported and scored as follows: 0 (no sexual activity) or 1–5. Specifically, “no sexual activity” in 12 items and “did not attempt intercourse” in 3 items represent a zero score. The remaining 4 questions (questions number 1, 2, 15, and16) do not carry a zero category. The zero categories were treated as the low point of the response scale for each item. Respondents could meaningfully rate their responses on a scale of 1–5 only when “sexual activity or intercourse” had occurred during the 4 weeks prior to the survey. In addition to sexual problems, there are various reasons for a woman to be sexually inactive for 4 weeks. Therefore, it is more appropriate to classify the zero categories as “not applicable” or “missing values.” The FSFI is designed as a universal screening instrument for samples that do not have such specific exclusion criteria; hence, we needed to be more selective for sexually active women after the sample data were obtained. Thus, we divided the CV-FSFI results obtained into 2 parts: sexually inactive women were excluded from all respondents, and then CV-FSFI scores of sexually active women were analyzed. Since all sexually inactive women were excluded, the study evaluated only the prevalence of sexual problems among sexually active women.

### Survey questionnaire

We used a questionnaire to investigate the demographic characteristics of the participants, such as age, body mass index (BMI), race, marital status, area, education, type of occupation, menstrual status, reproductive history, chronic-disease history, and gynecological disorders. Chronic diseases included hypertension, diabetes, stroke, bronchitis, cancer, and depression. Gynecological diseases included endometriosis, pelvic inflammatory disease, chronic pelvic pain, and myoma. Menopause status was defined using the criteria of the Stages of Reproductive Aging Workshop as follows: premenopausal, women having regular menses; and postmenopausal, no menses in the last 12 months.^19^ Pelvic surgery included hysterectomy, cesarean section, myomectomy, ovarian cyst stripping, and ectopic pregnancy surgery.

### Statistical analysis

Quantitative data with normal distribution were expressed as mean ± SD. Quantitative data with nonnormal distribution were represented by median and interquartile range. The classified data were represented as *n* (percentage). We used multiple linear regression models to evaluate the risk factors for sexual problems. A *P*-value of <0.01 was considered statistically significant. Analyses were compiled by a professional statistician using the IBM SPSS Statistics v.28 for Windows.

## Results

### Characteristics of the sample population

In this study, we included 5515 women with an active sex life during the previous 4 months (Figure 1). Data regarding the general characteristics of the included women are presented in Table 2. The mean age of the women was 39.76 ± 10.53 years, and the BMI was 23.38 ± 3.20 kg/m^2^. The results revealed that 44.81% of women were from rural areas; 0.51% were members of minority groups; 51.55% engaged in mental labor; 4.93% women were graduates of an educational institution; 97.72% were married; 67.25% had experienced spontaneous vaginal delivery; 35.14% were multiparous (≥ 2 births); 83.34% were premenopausal; 35.97% had pelvic surgery; 0.38% had spinal surgery; 15.23% had chronic disease, and 31.86% had gynecologic disease. The chronic diseases included hypertension (2.19%), diabetes (8.83%), stroke (2.27%), bronchitis (0.31%), cancer (1.49%), and depression (0.18%). The gynecological diseases included endometriosis (4.48%), pelvic inflammatory disease (31.77%), chronic pelvic pain (20.40%), and myoma (0.60%). The number of women with a history of smoking and drinking were 238 (4.32%) and 31 (0.56%), respectively.

### Results of CV-FSFI

The mean CV-FSFI total score for the initial entering analytical sample (*n* = 6720) of both sexually active and inactive women, was 21.64 ± 8.87 (99% CI, 21.43-21.85) (Table 1). The mean CV-FSFI score for only the sexually active women (*n* = 5515) was 25.38 ± 4.20 (99% CI 25.27-25.49). For any sexual problem reported, the lowest score was for sexual desire (3.24 ± 0.83) followed by arousal (3.71 ± 0.89), orgasm (4.04 ± 0.94), and satisfaction (4.19 ± 0.92). Many women were affected by more than 1 type of sexual problem.

**Table 1:** CV-FSFI in each domain.

Domain	*n* = 6720^a^		*n* = 5515^b^	
	Domain CV-FSFI (mean ± SD)	99% CI	Domain CV-FSFI (mean ± SD)	99% CI
Desire	2.94 ± 1.03	(2.91-2.97)	3.24 ± 0.83	(3.21-3.27)
Arousal	3.04 ± 1.63	(2.99-3.10)	3.71 ± 0.89	(3.68-3.74)
Lubrication	4.06 ± 2.06	(4.00-4.13)	4.95 ± 0.88	(4.92-4.98)
Orgasm	3.32 ± 1.77	(3.26-3.37)	4.04 ± 0.94	(4.01-4.08)
Satisfaction	3.97 ± 1.00	(3.94-4.00)	4.19 ± 0.92	(4.16-4.23)
Pain	4.31 ± 2.18	(4.24-4.38)	5.25 ± 0.93	(5.22-5.28)
Total score	21.64 ± 8.87	(21.36-21.92)	25.38 ± 4.20	(25.24-25.53)

CV-FSFI, Chinese version of the Female Sexual Function Index. ^a^Both sexually active and inactive women (*n* = 6720);

^b^Only sexually active women (*n* = 5515).

**Table 2:** General characteristics of women participants. (*n* = 5515).

Characteristics	*n* (%)	t/F	*P*-value
Age (years)	39.76 ± 10.53		
20-29 (ref.)	1109 (20.11)	557.700	<0.001
30-39	1909 (34.61)		
40-49	1364 (24.73)		
50-59	806 (14.61)		
60-69	327 (5.93)		
BMI (kg/m^2^), mean (SD)	23.38 ± 3.20		
Normal weight (18.5-23.9) (ref.)	3068 (55.63)	32.400	<0.001
Underweight (<18.5)	262 (4.75)		
Overweight (24-27.9)	1717 (31.13)		
Obese (≥28)	468 (8.49)		
Area			
Urban (ref.)	3044 (55.19)	6.301	<0.001
Rural	2471 (44.81)		
Race			
Han (ref.)	5487 (99.49)	−1.040	0.308
Minority	28 (0.51)		
Occupation			
Mental labor (ref.)	2843 (51.55)	13.798	<0.001
Physical labor	2672 (48.45)		
Education			
Less than high school (ref.)	457 (8.29)	176.327	<0.001
Junior high school graduate	1902 (34.49)		
Senior high school graduate	1026 (18.60)		
College graduate	2130 (38.62)		
Income level (yuan)			
≤2000 (ref.)	2508 (45.48)	36.071	<0.001
2000-4000	1949 (35.34)		
4000-6000	857 (15.54)		
>6000	201 (3.64)		
Marital status			
Married (ref.)	5389 (97.72)	23.998	<0.001
Single	100 (1.81)		
Divorced	20 (0.36)		
Widowed	6 (0.11)		
Delivery pattern			
Vaginal spontaneous delivery (ref.)	3709 (67.25)	144.377	<0.001
Nulliparous	484 (8.78)		
Vaginal assisted delivery	67 (1.21)		
Cesarean section	1255 (22.76)		
Parity			
Primiparous (= 1) (ref.)	3093 (56.08)	257.604	<0.001
Nulliparous	484 (8.78)		
Multiparous (≥2)	1938 (35.14)		
Maximum fetal weight			
Normal weight (ref.)	4433 (80.38)	70.095	<0.001
Nulliparous	484 (8.78)		
Less than normal weight	93 (1.69)		
Macrosomia	505 (9.16)		
Menstrual status			
Premenopausal (ref.)	4596 (83.34)	39.796	<0.001
Postmenopausal	919 (16.66)		
Pelvic surgery			
No (ref.)	3531 (64.03)	2.777	0.005
Yes	1984 (35.97)		
Spinal surgery			
No (ref.)	5494 (99.62)	1.059	0.302
Yes	21 (0.38)		
Smoking			
No (ref.)	5277 (95.68)	4.144	<0.001
Yes	238 (4.32)		
Drinking			
No (ref.)	5484 (99.44)	3.155	0.004
+Yes	31 (0.56)		
Chronic diseases			
No (ref.)	4675 (84.77)	18.108	<0.001
Yes	840 (15.23)		
Gynecologic diseases			
No (ref.)	3758 (68.14)	6.847	<0.001
Yes	1757 (31.86)		

Chronic diseases included hypertension, diabetes, stroke, bronchitis, cancer and depression; gynecological diseases included endometriosis, pelvic inflammatory disease, chronic pelvic pain and myoma. BMI, body mass index.

**Figure 2:**
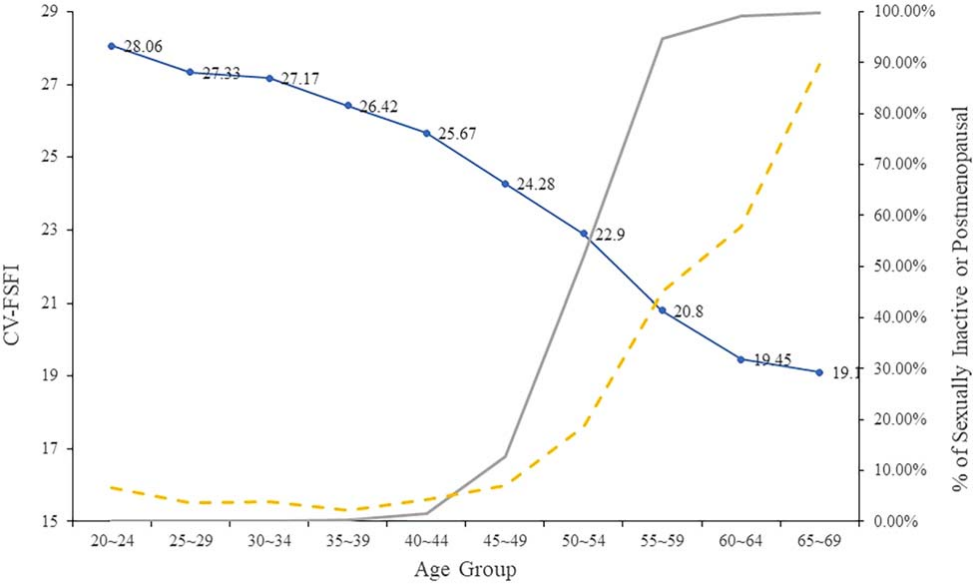
CV-FSFI, postmenopausal, and sexual inactivity across age groups. Blue line refers to CV-FSFI based on the sample of 5515 of sexually active women. Gray line refers to percentages of postmenopausal women. Yellow dashed line refers to percentages of sexually active women. These were based on the initial sample of 6720 sexually active and inactive women. CV-FSFI, Chinese version of the Female Sexual Function Index.

To study the differences in the CV-FSFI scores of different age groups, we plotted the variations in the CV-FSFI scores within different age groups (Figure 2). The results indicate that CV-FSFI scores decreased with age. Meanwhile, the proportion of postmenopausal and sexually inactive women increased with age.

### Factors associated with female sexual problems

**Figure 3:**
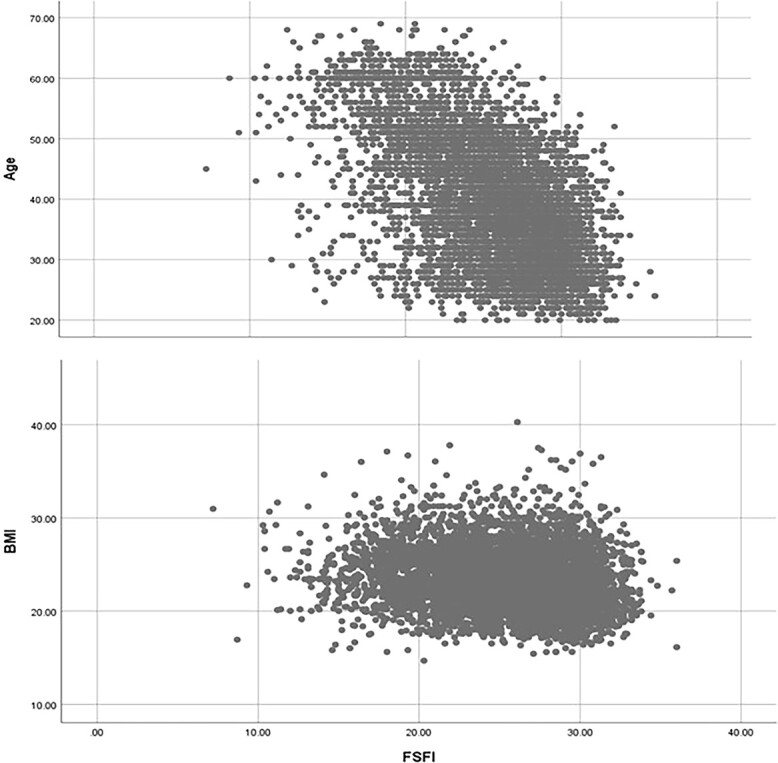
Scatter plot of age, BMI, and CV-FSFI. BMI, body mass index; CV-FSFI, Chinese version of the Female Sexual Function Index.

Table 3 and Figure 3 display the correlation analysis of CV-FSFI with age and BMI. There was a significant weak negative correlation between CV-FSFI and both age (r = −0.538, *P* < 0.001) and BMI (r = −0.152, *P* < 0.001).

**Figure 4:**
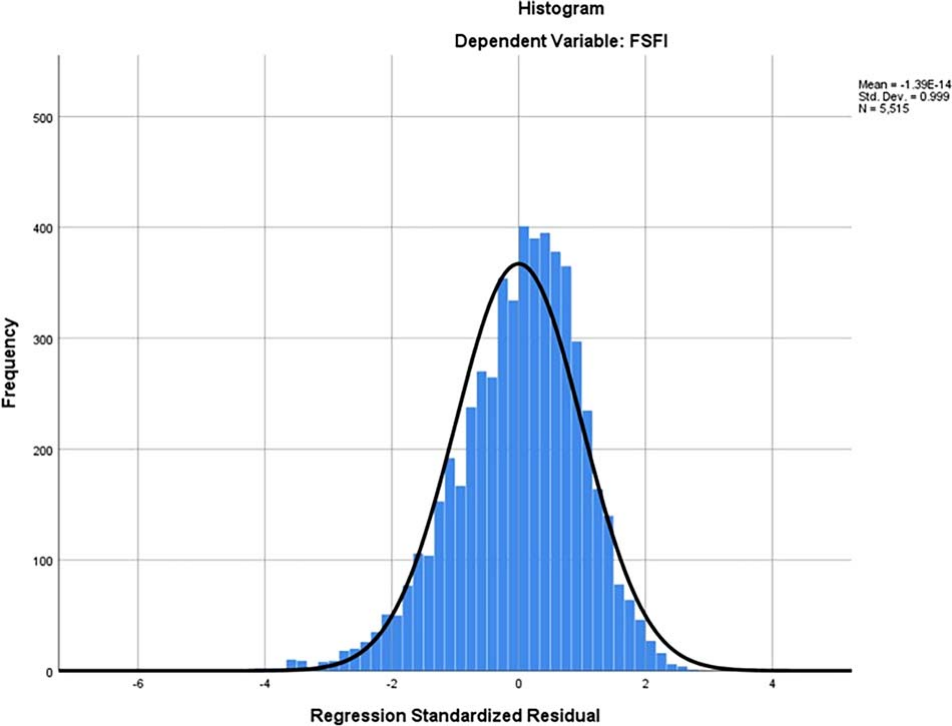
Regression standardized residual (1).

**Figure 5:**
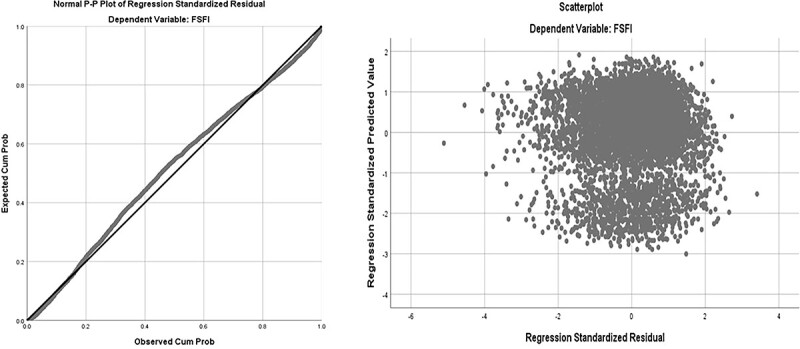
Regression standardized residual (2).

**Figure 6:**
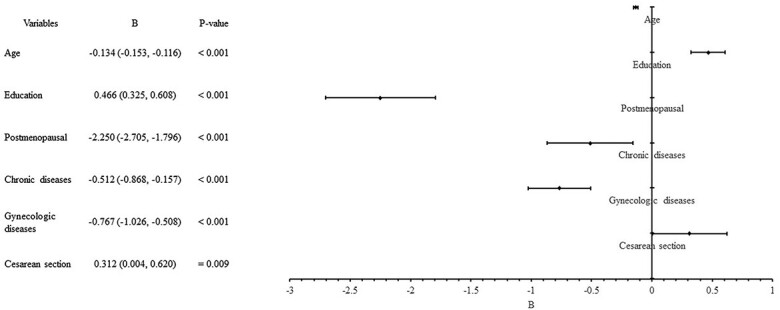
Forest plots of independent factors associated with CV-FSFI. BMI, body mass index; CV-FSFI, Chinese version of the Female Sexual Function Index.

Results of the multivariate linear regression analysis are shown in Table 5. In all, 16 variables, including age, BMI, area, occupation, education, income level, marital status, delivery pattern, parity, maximum fetal weight, menstrual status, pelvic surgery, smoking, drinking, chronic diseases, and gynecologic diseases were correlated with CV-FSFI. According to the multivariate linear regression analysis, 6 factors significantly affected the sexual function of the study participants. The independent variables adopted in the model were explained in 33% of the total volatility of the dependent variable (adjusted *R*^2^ = 0.33) (Table 4, Figure 4, and Figure 5). Figure 6 depicts the forest plots of independent factors associated with CV-FSFI. Negative numerical coefficients were found for model predictors of age (*B* = −0.134, *P* < 0.001), postmenopausal (*B* = −2.250, *P* < 0.001), chronic diseases (*B* = −0.512, *P* < 0.001), and gynecologic diseases (*B* = −0.767, *P* < 0.001). This clearly indicates that sexual problems depend on age, postmenopausal, chronic diseases, and gynecologic diseases. However, education (*B* = 0.466, *P* < 0.001) and cesarean section (*B* = 0.312, *P* = 0.009) were positive numerical coefficients, and hence prevent sexual problems.

**Table 3:** Relationship of CV-FSFI with age and BMI.

Characteristics	Mean ± SD	*r*	*P*-value
Age (years)	39.76 ± 10.53	−0.538	<0.001
BMI (kg/m^2^)	23.38 ± 3.20	−0.152	<0.001

BMI, body mass index; CV-FSFI, Chinese version of the Female Sexual Function Index.

Pearson correlation analysis was used to test the association of CV-FSFI and various indicators.

**Table 4:** Model summary of multivariate linear regression.

*R*	*R* ^2^	Adjusted *R*^2^	SEM	D-W
0.576	0.332	0.330	3.435	1.844

D-W, Durbin Watson test.

**Table 5:** Multivariate linear regression of CV-FSFI.

Variables	*B*	S.E.	ExP (*B*)	*P*-value	99% CI	
					lower limit	Upper limit
Age	−0.134	0.007	−0.337	<0.001	−0.153	−0.116
Education	0.466	0.055	0.114	<0.001	0.325	0.608
Postmenopausal	−2.250	0.176	−0.200	<0.001	−2.705	−1.796
Chronic diseases	−0.512	0.138	−0.044	<0.001	−0.868	−0.157
Gynecologic diseases	−0.767	0.101	−0.085	<0.001	−1.026	−0.508
Caesarean section	0.312	0.120	0.031	0.009	0.004	0.620

CV-FSFI, Chinese version of the Female Sexual Function Index.

*P* <0.01.

## Discussion

The present study is to our knowledge the first to evaluate the sexual function of women in Shanxi, China. Sexuality is a natural part of human life.^20^ Sex is the basis of human development and reproduction. Women undergo many critical stages in their lives and play a vital role in society. Because most women are reluctant to seek treatment as they tend to ignore their sexual status, they may remain underdiagnosed and undertreated. FSD adversely affects the quality of life of women. Recently, the field of sexual medicine has made great strides. Researchers have made remarkable progress in every area of investigation to provide better treatment to women who report sexual problems in daily practice.^21^ FSD is not easy to diagnose, because it is difficult to measure the clinical distress associated with sexual symptoms in women.^22–24^ The FSFI is a multidimensional scale that assesses sexual function in women. It is noteworthy that objective measures of sexual function do not reflect the subjective experiences of women themselves. Thus, FSFI can only be used to indirectly reflect a woman’s sexual function, with a lower score indicating a higher risk of FSD.

Previous studies reported that approximately 40%-45% of adult females encounter sexual problem at any time in life.^25^ Population-based studies in Asia have reported that the prevalence rates for FSD range from 26.1% to 73.2%.^26^ Shin et al. reported an incidence of 46.7% for FSD in Korea.^27^ While, Singh et al. reported a prevalence rate of 73.2% for FSD in South India.^28^ Earlier studies conducted in a large region of China reported a prevalence of 29.7% for FSD.^16^ Consistent with a previous study, we found that orgasm, desire, and arousal scores are the affected domains.^29^ Ojomu et al.^30^ reported that the proportions of sexual disorders were: 39% for desire disorder, 40% for arousal disorder, 31% for pain, and 55% for orgasmic disorder.

The complex interaction of many factors, such as psychological, social, and physiological concerns and relationships with partners, plays a role in the etiology of female sexual problems.^31^^,^^32^ Because sexually inactive women were excluded from this study, only the prevalence of sexual problems among sexually active women were evaluated in this study. Of the various risk factors for female sexual problems, many studies have focused on demographic characteristics.^33^ The conclusion of our study is that increasing age, postmenopausal, chronic diseases, and gynecological diseases are risk factors for sexual problems, whereas high education levels and childbirth via cesarean section were protective factors for sexual problems.

Advanced age has been reported as a significant risk factor for female sexual problems.^34^ Our results, like those of other studies worldwide, show that sexual problems worsen with age. With an increase in age, most women experience varying degrees of changes in sexual function, manifested primarily as a lack of sexual desire, decreased frequency of sexual activity, lubrication disorder, pain, and fear of sexual intercourse.^35^^,^^36^ Therefore, we need to provide health education to increase understanding of sexual function in elderly women and help them cope with the physical, psychological, and sexual problems they face.

Similar to previous studies, in the present study we identified postmenopausal status as a risk factor for female sexual problems.^34^^,^^37^ Due to decreased ovarian function and reduced estrogen levels, postmenopausal women are more likely to have symptoms of low sexual desire and only infrequent sex.^19^^,^^38^ Reduction of the effect of estrogen on the vaginal epithelium is results in relaxation of the pelvic floor muscles and reduction of vaginal folds. Decreased secretion from the vaginal glands leads to decreased lubrication. Therefore, we should pay attention to the effects of the aging process on the sexual health of women. It is necessary to conduct health education to create awareness and in women and improve their understanding of female sexual problemsin adult women along with encouraging beneficial activites such as muscle exercises to strengthen the pelvic floor. In addition, lubricant or estrogen can also be given according to the patient’s need to improve symptoms caused by dryness.

Education influences a woman’s lifestyle, living environment, and relationships with sexual partners. Women with high education levels tend to enjoy high social status and good sexual function, and these women are thus more likely to adjust to physical and mental conditions and family relations, have a better grasp their health, and have good coping and self-management abilities.^39^ These characteristics are linked to high education levels in women, which are often associated with liberal values and better knowledge. In contrast, Ibrahim et al.^34^ showed that the incidence of female sexual problems is high in better-educated women who have the ability to freely express their dissatisfaction.

Chronic diseases induce female sexual problems by altering hemodynamic changes and neuroendocrine regulation.^40^ Hypertension has a complex influence on the pathogenesis of female sexual problems, leads to decreased vaginal lubrication and orgasm, and increases pain during intercourse.^41^ Consistent with the existing literature, our results suggested that diabetes and cancer also affect sexual function.^42^^,^^43^ Due to poor blood sugar control, diabetic patients suffer from vascular neuropathy, which causes congestion of the vagina and its surrounding tissue, thus reducing the feeling of orgasm and hence affecting sexual function.^44–46^ Additionally, women with diabetes are less responsive to sexual stimulation. The pathological factors of cancer and stroke are mainly medical interventions and physical disorders, which reduce libido.^47–49^ Women suffer from various sexual disturbances and psychological problems with gynecological diseases due to their clinical manifestations, such as local itching, pain, and abnormal secretions.^50^^,^^51^ In the current study, we found that endometriosis, pelvic inflammatory disease, and chronic pelvic pain significantly reduce female sexual function. We assumed that the accompanying pain in such disorders makes sexual activity distressing and increases the fear of it. Therefore, women suffering from chronic and gynecological diseases should be able to receive high-quality medical care, including assessment of their sexual function so that , FSD is detected and treated early in this vulnerable population.

Murtagh J et al.^52^ reported that delivery causes sexual dysfunction by affecting various physical and psychological factors that interfere with sexual behavior. However, we found that cesarean section was a protective factor for sexual problems, probably because cesarean section reduces pelvic floor tissue damage during delivery. Needless to say, these areas should be more deeply studied in future research to improve comprehension and treatment of other factors associated with FSD.

## Limitations

Inevitably, our study has a few limitations. The age distribution of women included in this study was uneven, with fewer women >40 years. Sexual partners were not surveyed due to the size of the questionnaire. Sexual function is known to be associated with cultural differences, religious beliefs, morals, ethics, and other social factors. However, whether female sexual problems have such different associations remains to be further explored. Urinary incontinence and pelvic organ prolapse were not assessed in this study. Finally, a questionnaire was used to obtain data regarding the issues being investigated, and answers to these questions may have been subjective, and thus additional tools and documentation are probably needed for accurate assessment.

## Conclusion

In conclusion, similar to other studies conducted globally, our study found that increasing age, postmenopause, chronic diseases, and gynecological diseases were risk factors, whereas higher education and cesarean section were protective factors for sexual problems in Chinese women. It is important for medical researchers to ask women these questions and understand any factors that may affect women’s sexual problems.
